# Natural frequencies facilitate diagnostic inferences of managers

**DOI:** 10.3389/fpsyg.2015.00642

**Published:** 2015-06-22

**Authors:** Ulrich Hoffrage, Sebastian Hafenbrädl, Cyril Bouquet

**Affiliations:** ^1^Department of Organizational Behavior, Faculty of Business and Economics, University of Lausanne, Lausanne, Switzerland; ^2^International Institute for Management Development, Lausanne, Switzerland

**Keywords:** bayesian inference, updating beliefs, natural frequency, representation format, management, executives, applied business statistics

## Abstract

In Bayesian inference tasks, information about base rates as well as hit rate and false-alarm rate needs to be integrated according to Bayes’ rule after the result of a diagnostic test became known. Numerous studies have found that presenting information in a Bayesian inference task in terms of natural frequencies leads to better performance compared to variants with information presented in terms of probabilities or percentages. Natural frequencies are the tallies in a natural sample in which hit rate and false-alarm rate are not normalized with respect to base rates. The present research replicates the beneficial effect of natural frequencies with four tasks from the domain of management, and with management students as well as experienced executives as participants. The percentage of Bayesian responses was almost twice as high when information was presented in natural frequencies compared to a presentation in terms of percentages. In contrast to most tasks previously studied, the majority of numerical responses were lower than the Bayesian solutions. Having heard of Bayes’ rule prior to the study did not affect Bayesian performance. An implication of our work is that textbooks explaining Bayes’ rule should teach how to represent information in terms of natural frequencies instead of how to plug probabilities or percentages into a formula.

## Introduction

Twenty years ago, Gigerenzer and Hoffrage (1995) demonstrated that Bayesian inferences can be improved without instructing participants how to solve such Bayesian tasks. By providing the relevant information not in terms of probabilities, percentages, or relative frequencies, as it is usually done, but in terms of natural frequencies, the percentage of correct (i.e., Bayesian) inferences tripled, specifically, from 16 to 46%. What is a Bayesian inference task and what are natural frequencies? Consider the following example:

The Skiwell Manufacturing Company gets material from two suppliers. Supplier A’s materials make up for 30% of what is used, with supplier B providing the rest. Past records indicate that 15% of supplier A’s materials are defective and 10% of B’s material are defective. Since it is impossible to tell which supplier the material came from once they are in the inventory, the manager wants to know: What is the probability that material comes from supplier A given that it has been identified as defective?

If the question was “*What is the probability that material, randomly drawn from the inventory, comes from supplier A*,” then the answer would be easy: 30%. Since 30 and 70% are the base rates for the two suppliers, A and B, respectively, one could simply use these base rates when asked about the prior probability that material comes from suppliers A or B. Taking supplier A as a reference, these two probabilities will henceforth be referred to as *p(H)* and *p(–H)*, which is the standard notation for the probability that a hypothesized event will occur (or not), or whether a hypothesis is true (or not).

The term “prior” refers to the point in time before diagnostic information has been given. In the example above, such data (D) has indeed been observed—specifically, the material has been identified as defective. This information should be used to update the prior probability. Following this update, the best estimate that the material comes from supplier A is the posterior probability, *p(H*|*D*). It can be calculated using Bayes’ rule:

$$p(H\text{|}D)=\frac{p(H)p(D\text{|}H)}{p\left(H\right)p\left(D\text{|}H\right)+p\left(-H\right)p\left(D\text{|}-H\right)}$$

where *p(D|H)* stands for the probability that material is defective if it comes from supplier A (in the example above, this probability is given by the relative frequency of 15%), and where *p*(*D*|*–H*) stands for the probability that material is defective if it comes from supplier B (in the example above, 10%).

Previous research has shown that people have difficulties to infer the posterior probability from the prior probability and the two likelihoods, *p(D|H)* and *p(D|–H)* (in terms of signal-detection theory, these two likelihoods are referred to as hit rate and false-alarm rate, respectively; in medical terms, the hit rate is called sensitivity and the false-alarm rate is the complement of the specificity). In order to give the reader a better chance to experience some empathy with participants, we do not reveal the Bayesian solution to the Skiwell Manufacturing Company task at this point—but note that the task was even harder for the participants because they, unlike the reader, did not have Equation 1 at their disposal. Kahneman and Tversky (1972) concluded from their research that participants do not integrate the three pieces of information; they rather confuse the posterior probability, *p*(*H*|*D*), with the likelihood of the observed data if the prior hypothesis were true, *p*(*D*|*H*), and provide the latter as an answer when asked for the former. Kahneman and Tversky consider this confusion as an application of the representativeness heuristic—which Gigerenzer (1996), in turn, considers to be a re-description or a “one-word explanation” (p. 594; see also Gigerenzer and Murray, 1987). Using the representativeness heuristic amounts to ignoring the base rates, which Kahneman and Tversky (1972) demonstrated with a between-subjects design: The posterior beliefs of two groups of participants were indistinguishable even though these two groups received different base rates and should hence have different prior probabilities. The authors concluded that “In his evaluation of evidence man is apparently not a conservative Bayesian: he is not Bayesian at all” (p. 450). This “base-rate neglect” is one of the prime examples for a cognitive fallacy investigated in the “heuristics and biases” program (Kahneman et al., 1982), and Bar-Hillel (1980) stated that “the genuineness, the robustness, and the generality of the base-rate fallacy are matters of established fact” (p. 215).

This conclusion has been challenged by Gigerenzer and Hoffrage (1995) with a study in which they represented the information about base rate and the two likelihoods in terms of natural frequencies. Using this representation format, our task reads as follows:

The Skiwell Manufacturing Company gets material from two suppliers. Out of 1,000 items, supplier A delivers 300 and supplier B delivers the remaining ones. Past records indicate that 45 of the 300 items delivered by supplier A are defective and that 70 out of the 700 items delivered by B are defective. Since it is impossible to tell which supplier the material came from once they are in the inventory, the manager wants to know: How many of the items that have been identified as defective come from supplier A?

**FIGURE 1:**
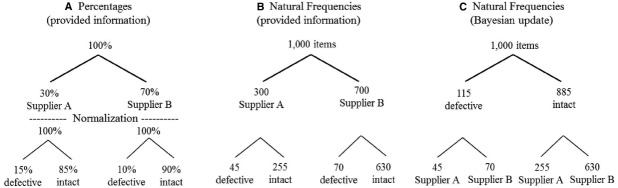
**Numerical information of the Skiwell Manufacturing Company task. (A)** Information provided in percentages. Hit rate and false-alarm rate have been normalized with respect to the base rates of the two suppliers. **(B)** Natural frequencies with suppliers at the intermediate level. The frequencies of the four conjunctive events implicitly contain the base rate information about the suppliers. **(C)** Natural frequencies with diagnostic information at the intermediate level. From the perspective of **(B)**, the tree in **(C)** represents a Bayesian update, in which the four distinct events are now conditioned on diagnostic information.

Natural frequencies are the frequencies that naturally result if a sample is taken from a population (or if the entire population is considered). In case of one hypothesis (H, with its complement –H) and one dichotomous, diagnostic variable that represents the data (D), natural frequencies are the four entries in the bivariate 2 × 2 table. The frequencies of the four conjunctive events can be displayed in two trees, in each of which the total sample size (or population) is the top node, and the four possible combinations are on the lowest level. One of these two possible trees displays the row margins at the intermediate level, and the other one the column margins. For instance, in Figure 1, Panel B, the two natural frequencies for a sample of 1,000 items are displayed at the intermediate level: 300 come from supplier A and 700 from supplier B, corresponding to the two base rates of 30 and 70%. From this tree in Panel B, it is relatively easy to determine the total number of defective items (45 + 70 = 115), and the total number of intact items (255 + 630 = 885). These two numbers are basically the margins of the diagnostic variable, and the first is included in the Bayesian solution to our task: Of the 115 defective items, 45 were delivered by supplier A. This is also the Bayesian response that we withheld above when we presented the problem in terms of percentages: *p(H|D)* = 0.39 (or, as a ratio, 45/115). From Figure 1, Panel B, it is also easy to construct the tree displayed in Panel C, which would also allow one to answer to other questions, for instance, how many of the intact items were delivered by supplier B.

The tree in Panel A displays the information as it has been represented in the initial version of the Skiwell Manufacturing Company task. What made it hard to derive the solution from this representation, compared to a natural frequency representation, was the fact that the two likelihoods have been normalized with respect to the base rates, and for exactly this reason, Gigerenzer and Hoffrage (1995) predicted that representations in terms of probabilities, percentages, and relative frequencies will not differ with respect to Bayesian performance (Prediction 4, p. 692). For a more detailed discussion of the notion of natural frequencies and its relationship to other representation formats, see Hoffrage et al. (2002), Gigerenzer and Hoffrage (2007), and Johnson and Tubau (in review).

Natural Frequencies have proven to facilitate diagnostic inferences in laypeople (Gigerenzer and Hoffrage, 1995), advanced medical students and advanced law students (Hoffrage et al., 2000), patients (Garcia-Retamero and Hoffrage, 2013), and physicians (Hoffrage and Gigerenzer, 1998). This result is well established (Mandel, 2015), it has been replicated by many others (e.g., Akl et al., 2011; Woloshin and Schwartz, 2011), and this work has received wide attention in the medical field and beyond (Gigerenzer, 2002, 2014; Gigerenzer et al., 2007; Gigerenzer and Gray, 2011). For a discussion about when and why natural frequencies are effective, see Brase (2008), Brase and Hill (2015), Gigerenzer and Hoffrage (2007), Hill and Brase (2012), and Johnson and Tubau (in review).

Bayesian inference problems are also vital to management decisions. For instance, a sales manager may be interested in whether a customer places more weight on quality than price if her yearly income is above average, a bank may be interested in whether it will see the annuity for a mortgage if the customer will lose his job, a project manager may be interested in whether the group will be able to complete the project in time if one of the key engineers will get sick unexpectedly, and so on. The fact that the task of updating beliefs is ubiquitous and also relevant in the world of business makes it even more surprising that, to the best of our knowledge, there is no research investigating whether natural frequencies are also beneficial for managers and management problems. This is exactly the aim of the present paper. The participants in the studies reported below were executives and business students who had to work on four different tasks with business-related content.

## Materials and Methods

### Participants

Participants were undergraduates at a business faculty (*n* = 259) and executives (*n* = 181; for a total *n* of 440). The undergraduates were either students enrolled in their third year of the Bachelor of Science in Management program of a public Swiss university who took the lecture “Judgment and Decision Making” of the first author, or students enrolled in their first year of the Master of Science in Management program who took the seminar “Analytic and Intuitive Judgment” of the second author. Over 3 years, three cohorts of bachelor students and in the fourth year, one cohort of bachelor students and one cohort of master students were tested, with 74, 45, 49, 62, and 29 students responding to the questionnaire. Demographic information was only collected for the last two cohorts: The bachelor students were on average 21.4 years old (SD = 1.2) and 51% were female, and the master students were on average 24 years old (SD = 1.9) and 30% were female. The entire population of the three earlier cohorts of bachelor students was demographically similar to the bachelor students of the last cohort.

The executives were also tested in a classroom setting, namely in their role as students in an executive MBA program. In fact, they enrolled in either of two different programs. One was the Executive MBA program offered by a public Swiss university in which they took a course “Managerial Decision Making and Negotiation” of the first author. Four different cohorts from four different years have been tested, with 27, 28, 22, and 43 respondents (*n* = 120). Average age was 38, 38, 37, and 38 years, and 87, 83, 72, and 76% were male. These participants are henceforth referred to as junior managers. The other program (Program for Executive Development, in fact a very prestigious and competitive program) was offered by a private Swiss business school. The executives took a course taught by the third author who had invited the first author as a guest lecturer. Two cohorts of the same module have been tested, with 27 and 34 participants each (*n* = 61). With an average age of 42 years, these executives were older than the ones from the public university, and so they are henceforth referred to as senior managers. In fact, many of them were directors or vice-presidents in their companies.

### Materials, Design, and Procedure

Four tasks have been used, all adapted from Groebner et al. (2007). The Skiwell Manufacturing task introduced above was one of them, the three others involved error/fraud detection (IRS Audit), success in the context of an auction (Techtronics Equipment), and quality control (Varden Soap). For each task, two versions were constructed, one in which the information was presented in percentages and one in which natural frequencies were used (see the Appendix for the exact formulations of these other three tasks, and Table 1 for the numbers involved in all four tasks).

**TABLE 1:** **The four tasks used in the present study with the information provided and the Bayesian solution**.

**Task**	**Condition**	**Base rate**	**Hit rate**	**False-alarm rate**	**Bayesian solution**
Skiwell manufacturing	Percentages	30	15	10	39.13
	Natural frequencies	300 of 1000	45 of 300	70 of 700	45 of 115
IRS Audit	Percentages	20	30	10	42.86
	Natural frequencies	200 of 1000	60 of 200	80 of 800	60 of 140
Techtronics equipment	Percentages	60	70	50	67.74
	Natural frequencies	60 of 100	42 of 60	20 of 40	42 of 62
Varden soap	Percentages	60	5	10	42.86
	Natural frequencies	600 of 1000	30 of 600	40 of 400	30 of 70

Each questionnaire consisted of two different tasks, either two percentage versions, or two natural frequency versions. Which task was paired with which other task and their order within the same questionnaires was counterbalanced, so that the number of respondents per task and per version was, ideally, equally distributed (minor deviations from an equal distribution were due to the fact that the number of students in a classroom was rarely divisible by the minimal number of questionnaires that would allow for an equal distribution, resulting in 111, 110, 111, 110 for the percentage versions, and 110, 108, 111, 109 for the frequency versions of Skiwell, IRS, Techtronics, and Varden Soap, respectively).

Students were given 7 min to work on the tasks. They were allowed to take notes. Some students had a pocket calculator with them (or a smartphone with this function), and very few asked whether they could use them. The answer was positive, but with respect to those who did not have one at their disposal, it was added that writing down a mathematical operation, for instance, a ratio, would be sufficient. In other words, we made it clear that we were not interested in whether they could enter numbers into a pocket calculator, but whether they were able to figure out *which* numbers to enter, and that writing down the correct operation would be treated as a correct response even if they would not convert it into an exact decimal. After 7 min the questionnaires were collected, but it could not have been prevented that some students continued writing during the collection procedure.

This procedure was slightly altered for the 62 bachelor students and the 29 master students who were tested during the last year of data collection. After 7 min, they were prompted to turn the questionnaire to a new page that was not included in the questionnaire of the 181 executives and the other 168 undergraduates. On this page, they entered their demographics and responded to several questions concerning their prior knowledge about Bayes’ rule. To prevent participants from being exposed to the term “Bayes’ rule” within the questionnaire before finishing the inference problems, these questions were only displayed via a projector once all students had finished working on the inference problems.

After having turned in their questionnaires, students received a lecture about Bayesian inferences and representation formats—sometimes right after the questionnaires, sometimes in another lecture. When those participants whose booklet did *not* include the page with the questions regarding Bayes’ rule were asked, during this debrief, whether they were familiar with this kind of task and whether they had received some instructions or training beforehand, for instance, in a lecture on statistics, very few (about 5% of the executives and about 10% of the management undergraduates) raised their hand.

### Analysis

The analysis was mainly based on outcomes, that is, on participants’ numerical responses. Following Gigerenzer and Hoffrage (1995), a response has been classified as Bayesian if the absolute difference between this response and the Bayesian solution was lower than one percentage point. This criterion was lenient enough to also include rounding up or down to the next whole number. In fact, many participants in the percentage condition were able to derive the Bayesian solution, wrote down the formula, used the pocket calculator of their smartphone to compute the exact value, but then wrote, for the Skiwell Manufacturing task, 39 or 40% (instead of 39.1304%).

Traces of cognitive processes, that is, notes and remarks that revealed how participants arrived at their answers, were also considered. If a participant provided a numerical response that we would have classified as Bayesian, but if the notes made it clear that this match was only coincidental and resulted from a non-Bayesian rationale, then we did not classify the response as Bayesian. Conversely, if a participant in the percentage condition wrote down a ratio that corresponded to the Bayesian solution, but did not compute the exact number (by hand or with a pocket calculator), we nevertheless classified it as a Bayesian answer—and as we already mentioned above, participants were informed about this.

## Results

### Do Natural Frequencies Facilitate Bayesian Inferences in Our Four Tasks?

**FIGURE 2:**
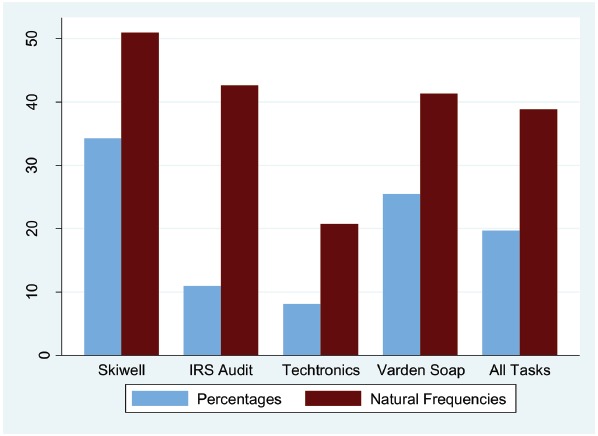
**Percentages of Bayesian responses, depending on whether the numerical information has been communicated in terms of percentages or natural frequencies**.

Yes. Figure 2 displays the percentages of Bayesian responses, both for the four tasks separately and across all tasks. In the percentage condition, 87 of 442 responses (19.7%) were Bayesian, and in the natural frequency condition, these were 170 of 438 (38.8%). A logistic regression confirmed that the format in which information was presented had a significant effect (*B* = 1.04, SE = 0.20, *z* = 5.24, *p* < 0.001; after controlling for task, order, and participant sample, and with standard errors clustered for each participant).

### Does Representation Format also Affect the Non-Bayesian Inferences?

**FIGURE 3:**
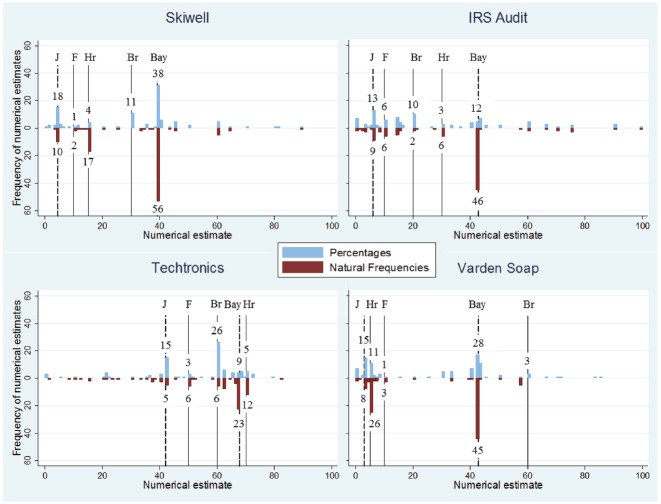
**Distribution of numerical estimates for the four tasks.** The three straight lines indicate information that has been given in the task: Br indicates the base rate for the focal category, Hr indicates the hit rate (that is, diagnostic information conditioned on the focal category, *p(D*|*H)*), F indicates the false-alarm rate (that is, diagnostic information conditioned on the non-focal category, *p(D*|*–H)*). The two dotted lines indicate possible ways of combining this information: Bay indicates the Bayesian solution, p(*H*|*D*), and J stands for Joint Occurrence of D and H, *p*(*D* and *H*). The numbers on these lines (and those on the y-axes) denote the frequencies of the corresponding numerical estimates. For instance, 10 participants in the percentage condition of the IRS task provided a numerical estimate between 20 and 20.99% (in fact, all these 10 participants wrote exactly 20%, which was identical to the base rate of that task), and 12 participants provided an estimate that has been coded as a Bayesian response (three gave the exact Bayesian response, either as the ratio 45/115, or they wrote down the exact number including the decimal, 42.86%, most likely with the help of a pocket calculator, one responded with 42.8%, and one with 42.9%. These five responses are displayed in the bracket ranging from 42.0–42.99%. The remaining seven participants responded with 43%. These seven estimates are displayed in the adjacent bracket, namely 43.0–43.99%, but they were nevertheless coded as Bayesian because our classification criterion allowed for rounding within one percentage point, see above). Note that something similar could be observed for each of the four tasks: the responses that have been classified as Bayesian are spread across two adjacent brackets, and hence the number of Bayesian responses is not visualized by one single bar, but rather consists of two lower numbers visualized by two bars.

While the previous analysis focused on the percentages of Bayesian responses, it is also useful to take a look at the full distribution of numerical estimates, independent of whether participants succeeded in deriving the Bayesian solution or not. Figure 3 provides such a more fine-grained picture of the distribution of numerical estimates for the four tasks. Two estimates in the natural frequency condition for which the numerator was larger than the denominator, and one of 125% in the percentage condition were classified as non-Bayesian in Figure 2, but were not graphically displayed in Figure 3.

Overall, most estimates were too low: Across all tasks in the percentage condition, 58.4% of the responses were lower than the Bayesian solution, 19.7% were classified as Bayesian, 21.7% were higher than the Bayesian solution and 0.2% were above 100%. For the natural frequency condition, these numbers were 43.4, 38.8, 17.4, and 0.4% respectively. When information has been presented in terms of natural frequencies, the responses were not only more often correct (see Figure 2), but also closer to the Bayesian solution: The average absolute difference between responses and Bayesian solution was 19.2 in the percentage condition, and 15.1 for natural frequencies (excluding responses above 100%). Regression analysis revealed that this difference was significant (*B* = 3.84, SE = 1.35, *t* = 2.84, *p* = 0.005; after controlling for task, order, and participant sample, and with standard errors clustered for each participant). Closer inspection, however, revealed that this difference was mainly due to the Bayesian responses. After these have been excluded, the picture even reversed: In the percentage condition, the average absolute difference of the remaining cases was 24.5, and in the natural frequency condition, it was 26.3 (but this effect was not significant: *B* = 2.12, SE = 1.27, *t* = 1.68, *p* = 0.095). In sum, in each of the two experimental conditions, most responses were too low. Participants’ responses were closer to the Bayesian solution in the natural frequency condition, but this effect was mainly due to the fact that there were more Bayesian responses in the first place.

Figure 3 also shows that most numerical estimates were either identical to one of the pieces of information that has been given for a particular task, namely the base rate (Br), the hit rate (Hr), or the false-alarm rate (F), or that they matched the Bayesian response (Bay) or the probability that D and H occur together (joint occurrence, J, which is the product of hit rate times base rate of focal hypothesis). Results from a more detailed analysis of the most frequently used cognitive strategies—indicated by the lines in Figure 3—will be reported in the next section that focusses on the effects of participant sample (Table 2).

**TABLE 2:** **Use of cognitive strategies, split by representation format and participant sample**.

						**Logistic regression results**					
	**Percentages**		**Natural frequencies**			**Format**		**Sample**		**Format × Sample**	
**Cognitive strategy**	**Undergraduates**	**Executives**	**Undergraduates**	**Executives**	**Total**	***B***	***p***	***B***	***p***	***B***	***p***
Bayesian	14.57	26.6	40.53	36.21	29.2	1.47	<0.001	0.22	0.39	1.0	0.012
Base rate	11.42	11.17	1.52	2.3	6.6	–2.21	0.001	0.47	0.55	–0.47	0.587
Hit rate	4.72	5.85	13.26	14.94	9.5	1.15	0.001	0.12	0.69	0.05	0.886
False-alarm rate	1.97	3.19	4.17	3.45	3.2	0.78	0.174	0.18	0.76	0.7	0.413
Joint occurence	12.6	15.43	4.55	11.49	10.6	–1.11	0.009	1.0	0.02	–0.77	0.158
Total observations	254	188	264	174	880						

The coefficients (B) and p-values result from five different logistic regressions, one for each strategy, that were conducted to determine how representation format and participant sample affected strategy use (after controlling for task and order, and with standard errors clustered for each participant). Total observations refer to the total number of responses on which the percentages reported in the cells are based, that is, the numbers in the cells denote column percentages.

### Who Performed Better: The Undergraduates or the Executives?

**FIGURE 4:**
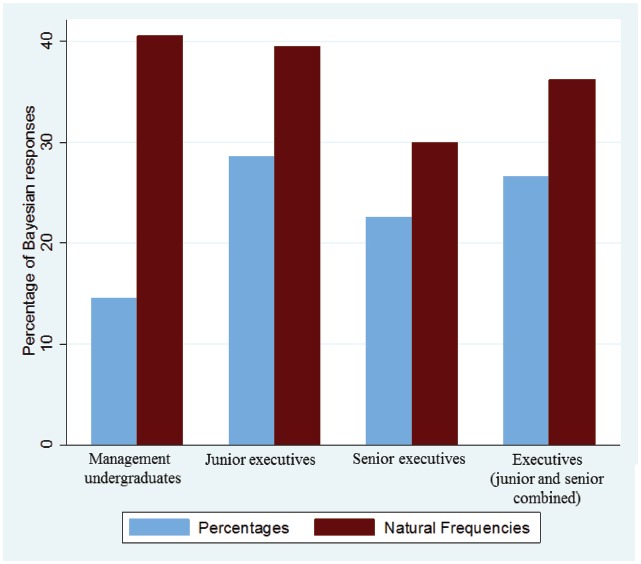
**Percentages of Bayesian responses, separately for the different groups of participants**.

Figure 4 displays the percentages of Bayesian responses for the different types of participants. No clear picture emerged. While the undergraduates performed worse than the executives in the percentage condition (14.6, 28.6, 22.6, and 26.6%, for undergraduates, junior executives, senior executives, and executives combined, respectively), they outperformed the executives when the information was represented in natural frequencies (40.5, 39.5, 30.0, and 36.2%, respectively).

The main effect of participant sample was not significant (*B* = 0.219, SE = 0.26, *z* = 0.86, *p* = 0.392), but the interaction between participant sample and representation format was (*B* = 0.99, SE = 0.40, *z* = 2.52, *p* = 0.012; after controlling for representation format, task, and order, and with standard errors clustered for each participant). Analyzing the contrast between undergraduates and executives (junior and senior combined) separately, revealed a significant difference in the percentage condition (14.6 vs. 26.6%, *B* = 0.796, SE = 0.310, *z* = 2.57, *p* = 0.010), while the difference in the natural frequency condition was negligible and not significant (40.5 vs. 36.2%, *B* = 0.213, SE = 0.252, *z* = 1.12, *p* = 0.398). Finally, analyzing the contrast between the two representation formats revealed a significant difference between the percentage condition and the natural frequency condition within the undergraduate sample (14.6 vs. 40.5%, *B* = 1.48, SE = 0.28, *z* = 5.32, *p* < 0.001), while the superiority of natural frequencies did not reach statistical significance within the sample of executives (junior and senior combined; 26.6 vs. 36.2%, *B* = 0.47, SE = 0.28, *z* = 1.67, *p* = 0.095).

Table 2 completes the picture by also including the non-Bayesian strategies. While Figure 3 splits the frequencies of the five different cognitive strategies according to representation format and task, Table 2 splits them according to representation format and participant sample (again, undergraduates vs. executives; the latter with junior and senior combined). This table also displays, for each strategy separately, the coefficients (*B*) and the *p*-values of the five different logistic regressions, each with the main effect of representation format and participant sample, and the interaction between representation format and sample (after controlling for task and order, and with standard errors clustered for each participant). For each of the five cognitive strategies, except for providing the false-alarm rate as response, the number of participants who provided the corresponding numerical estimate significantly differed between the percentage condition and the natural frequency condition. In contrast, for none of the strategies, except for joint occurrence, we observed a significant effect of participant sample, and for none of the strategies, except for Bayesian, the interaction between representation format and participant sample reached significance.

### Did the Order Matter?

No. Across all tasks, participants, and both representation formats, a response has been classified as Bayesian in 30.5% for tasks on the first page of the questionnaire and 28.0% for tasks on the second page (in a logistic regression, the difference was not significant; *B* = 0.131, SE = 0.115, *z* = 1.14, *p* = 0.254; after controlling for representation format, participant sample and task, and with standard errors clustered for each participant).

### Did Prior Knowledge of Bayes’ Rule Make a Difference?

For none of our executive participants, but for 91 of our 259 undergraduate participants (62 bachelor and 29 master students) the booklet contained questions on demographics and on prior knowledge about Bayes’ rule. A majority of 46 (74%) of the bachelor students and 17 (59%) of the master students responded that they have heard of Bayes’ rule before this lecture (overall, 63 of 91 = 69%). With the exception of one bachelor student, all of those who had heard about Bayes’ rule said it was taught to them in a course: 35 (56%) at school and 27 (44%) at the university (62/91 = 68%). A minority of 27 (44 %) of the bachelor students and 13 (45%) of the master students responded that they know when Bayes’ rule is applicable, and 7 (11%) of the bachelor students and 11 (38%) of the master students (18/91 = 20%) were able to provide a short and correct explanation (we applied a very lenient criterion and coded answers such as “when conditional probabilities need to be computed” as correct). When asked whether they are able to formulate Bayes’ rule, 21 (34%) of the bachelor students and 8 (28%) of the master students (29/91 = 32%) responded with yes, but only 10 (16%) of the bachelor students and 1 (3%) of the master students (11/91 = 12%) wrote down the correct formula. We should add that none of these 11 students reproduced our Equation 1 exactly, instead they all used an abridged version and wrote *p*(*H*|*D*) = *p*(*H*)*p*(*D*|*H*)/*p*(*D*)—which we coded as correct, despite of the fact that we cannot exclude the possibility that someone used a smartphone with internet access, and despite having doubts that someone who wrote down this abridged version was able to use it for our Bayesian tasks and to understand that the denominator, *p*(*D*), amounts to *p*(*H*)*p*(*D*|*H*) + *p*(*–H*)*p*(*D*|*–H*). These doubts lead straightforward to our next question: How have these differences between students been reflected in their ability to produce Bayesian responses?

To the extent that teaching and instructions leave traces, one may expect that those who had heard of Bayes’ rule performed better than those who had not. To test this hypothesis, we included the responses to each of the four questions about prior knowledge of Bayes’ rule as a predictor of performance in a separate logistic regression, controlling for format, task, order, and participant sample, and with standard errors clustered for each participant. None of these regressions revealed a significant effect of prior knowledge on performance in our Bayesian tasks. To investigate whether prior knowledge affects the ability to produce Bayesian responses differentially, dependent on the format of the question, we reran the logistic regressions, this time with an additional interaction term between participants’ responses and representation format. The result was the same: for none of the four questions concerning prior knowledge of Bayes’ rule was there a significant main effect or a significant interaction effect on Bayesian performance.

## General Discussion

To the best of our knowledge, the present study is the first to test whether natural frequencies facilitate Bayesian reasoning with management related tasks given to management undergraduates and executives. Even though the effect was not as strong as in previous studies, it is still larger than most effects observed in the social sciences: About twice as many participants came up with the Bayesian response when information was presented in terms of natural frequencies compared to percentages.

### Distribution of Bayesian and Non-Bayesian Responses: Toward an Ecological Analysis of Bayesian Inference Tasks

A remarkable finding of our study is that most non-Bayesian estimates were lower than the Bayesian solution (Figure 3). This pattern is unusual, at least when compared to information in typical medical diagnostic tasks in which the Bayesian response is usually low and participants’ responses are usually much higher (e.g., Eddy, 1982). How could one account for the different response patterns? One obvious dimension along which the tasks vary is numbers used for each particular problem. For most diseases, the base rate is relatively low, and for most diagnostic tests in medicine, the hit rate (or sensitivity) is relatively high, and the false-alarm rate is relatively low. This was different in our four tasks (see Table 1), for which the base rates were—compared to most medical tasks—higher, the hit rates were lower, and the false-alarm rates were higher. Hence, it seems to be straightforward to explore the extent to which the base rate, the hit rate, and the false-alarm rate affect strategy use. When we started to do exactly this, it soon became evident that a larger database would be extremely useful, and so we also included the responses to the fifteen tasks of Gigerenzer and Hoffrage (1995) in the analysis. Moreover, we complemented the set of the three quantitative task variables with three qualitative dimensions—norm deviation, stakes, and main focus—and subsequently used these task characteristics to account for the variance of participants’ responses and strategy use. This investigation, which can be considered as an example of an ecological analysis of Bayesian inferences, goes way beyond the scope of the present paper, and hence we report the results elsewhere (Hafenbrädl and Hoffrage, in review).

### Differences between Undergraduates and Executives

We do not know why undergraduates outperformed the executives when information was presented in terms of natural frequencies, whereas executives outperformed the undergraduates when information was presented in terms of percentages. Formulating this finding as an interaction, though, may help to find a possible explanation. While representation format played a larger role for undergraduates, executives were relatively immune against this manipulation (Figure 4). In fact, within the sample of executives the effect of representation format (differences of 9.6 percentage points in favor of natural frequencies) did not reach significance (*p* = 0.095, which may, of course and as always for non-significant differences, be an issue of statistical power). There might be two ways to arrive at a response: arithmetic calculation and intuitive estimation. Maybe executives had a more intuitive approach, possibly based on their experience with similar problems in the world of business (Klein, 2002). If such experience is used, then representation format might indeed play less of a role. In contrast, undergraduates lack such experience and are hence more likely to approach the tasks with logic, reasoning, and arithmetic. The fact that natural frequencies facilitate the computation (Gigerenzer and Hoffrage, 1995) may hence account for the fact that undergraduates benefitted quite a lot from this representation—more than the executives did. But we must admit that this consideration is highly speculative and we should add that we did not find a similar pattern when comparing medical students (Hoffrage et al., 2000) to experienced physicians (Hoffrage and Gigerenzer, 1998).

### Order Effects: Time Pressure and Training

More participants came up with the Bayesian response for tasks on the first page compared to tasks on the second page (difference of 2.5 percentage points). There are two main explanations for order effects: time pressure and training. If time pressure played a role, then we should expect that performance declines. In fact, out of those participants who provided an estimate to only one task, there were 28 who did so for the task on the first page, and 12 who did so only for the task on the second page (corresponding to a difference of 4.8 percentage points). In contrast, if training effects played a role, then we should expect that performance will increase. To the extent that the observed effect (2.5% better performance on first page) can be conceived as a result of a simple linear combination of the two possible components, time pressure and training, the effect of training is probably larger than the 2.5% that we observed, simply because this difference of 2.5% might have been overshadowed by the effect of time pressure. But we hasten to add that the observed difference was miniscule and of minor importance from a theoretical and practical point of view, and that our data does not allow us to assess the two possible contributing effects independently.

### Teaching Bayesian Inferences

Another remarkable finding of the present study is that 69% of the undergraduates whom we asked said that they had heard of Bayes’ rule, 68% said that they had been taught about it, 40% said they knew when it is applicable, 20% were actually able to correctly specify this, 32% said they were able to formulate Bayes’ rule, and only 12% could actually do so (and even this number must probably be corrected downwards, see result section). These numbers suggest that one should not be too optimistic that teaching Bayes’ rule leads to sustainable knowledge, retrievable from long-term memory. As one of the physicians studied in Hoffrage and Gigerenzer (1998) remarked to the experimenter after having filled out the questionnaire: “We have learned a formula at university, but I have forgotten it.” Moreover, none of the variables concerning prior knowledge of Bayes’ rule had a significant effect on Bayesian performance in our task. This lack of relationship could well be due to lack of statistical power, but if 91 participants are not enough to establish any relationship then such an effect, if existing at all, may be too small to be of practical importance.

Why is it that prior exposure to Bayes’ rule seems to make almost no difference? We suspect that difficulties to remember Bayes’ rule and to benefit from instructions may be related to how it is taught. In fact, inspecting an informal sample of textbooks on business statistics (Lawrence and Pasternack, 2002; Anderson et al., 2010; Newbold et al., 2010; Taylor, 2010) revealed the same picture as for the medical field: Bayes’ rule is taught almost exclusively using probabilities. We agree that such textbooks must ensure that a student will, at the end of the lessons, be able to handle probability information, but we disagree that the best way to get there is to teach how to insert probabilities into Bayes’ rule. Instead, we propose that students should be taught how to convert probabilities into natural frequencies. Sedlmeier and Gigerenzer (2001) have shown that a computerized implementation of such training is by far more effective compared to traditional rule training: the proportion of accurate answers doubled when participants had learned to represent probabilities as natural frequencies, as opposed to inserting them into Bayes’ rule. Kurzenhäuser and Hoffrage (2002) obtained similar results in a classroom setting with medical students and diagnostic tasks from human genetics. Note that in both studies, the success of the two treatments—in one, students had been taught how to plug in probabilities into Bayes’ rule, and in the other, they had been taught how to convert probability information into natural frequencies and derive the solution from there—has been evaluated by giving participants tasks with information presented in terms of probabilities. Taken together, the findings presented in this paper—supported by Sedlmeier and Gigerenzer’s (2001) and Kurzenhäuser and Hoffrage’s (2002) evaluation of tutorial programs—suggest that textbooks should no longer teach how to plug probabilities or percentages into a formula but rather instruct how to represent information in terms of natural frequencies to achieve a more sustainable mastery of Bayesian inference tasks.

Updating beliefs is a vital task, also in the domain of management. Representing information in terms of natural frequencies reduces computational complexity, improves understanding and boosts Bayesian performance. The posterior probability that managers can also benefit from natural frequencies, given the data of the present study, has definitely increased compared to the prior.

### Conflict of Interest Statement

The authors declare that the research was conducted in the absence of any commercial or financial relationships that could be construed as a potential conflict of interest.

## Appendix

Sentences or fragments of sentences that appear in parentheses and start with “P:” were only used in the percentage version, and those starting with “F:” were only used in the natural frequency version.

### IRS Audit

This year experts project that (P: 20% of all) (F: 200 out of 1000) taxpayers will file an incorrect tax return. To identify such incorrect returns, the Internal Revenue Service (IRS) has been implemented. Unfortunately, this service is not perfect. IRS auditors detect an error for (P: 30% of the) (F: only 60 of those 200) tax returns that are incorrect, and it will indicate an error in (P: 10% of the) (F: 80 of these 800) tax returns that are correct.

The IRS has just notified a taxpayer there is an error in his return.

(P: What is the probability that the return actually has an error? _____ %)
(F: How many of the tax payers who have been notified by the IRS that there is an error in their return, do actually have an error? _____ of _____)

### Techtronics Equipment Corporation

The Techtronics Equipment Corporation has developed a new electronic device that it would like to sell to the US Military for use in fighter aircraft. The sales manager knows that the military has placed an order (P: in 60% of) (F: in 60 of 100) similar cases. After making an initial sales presentation, military officials will often ask for a second presentation to other military decision makers. Historically, (P: 70% of successful companies are asked to make a second presentation, whereas only 50% of unsuccessful companies are asked back a second time.) (F: in 42 of the 60 successful cases, the companies were asked to make a second presentation, whereas for the unsuccessful cases, the companies were asked back a second time in only 20 of the 40 cases.)

Suppose Techtronics Equipment has just been asked to make a second presentation and so the sales manager wonders:

(P: What is the probability that the company will make the sale? _____ %)
(F: In how many of the cases in which a company has been called back, did this company receive an order? _____ of _____)

### Varden Soap Company

The Varden Soap Company has two production facilities, one in Ohio and one in Virginia. The company makes the same type of soap at both facilities. (P: The Ohio plant makes 60% of the company’s total soap output, and the Virginia plant 40%.) (F: Imagine 1000 containers of soap. The Ohio plant produces 600 of these containers, and the Virginia plant produces 400.) All soap from the two facilities is sent to a central warehouse, where it is intermingled. After extensive study, the quality assurance manager has determined that (P: 5% of the soap produced in Ohio and 10% of the soap in Virginia is) (F: 30 of the 600 soap containers produced in Ohio and 40 of the 400 soap containers produced in Virginia are) unusable due to quality problems. When the company sells a defective product, it incurs not only the cost of replacing the item but also the loss of goodwill. The vice president for production would like to allocate these costs fairly between the two plants.

(P: To do so, he wants to know, for instance: What is the probability that a soap was produced in Ohio given that it is defective? _____ %)
(F: To do so, he wonders, for instance: How many of the container with defective soap where produced in Ohio? _____ of _____)
